# UKFPO F2 Standalone: A brief guide

**DOI:** 10.1016/j.amsu.2022.104594

**Published:** 2022-09-13

**Authors:** Ikechukwu Chukwudi

**Affiliations:** LIFT F2 Standalone Trainee, Wirral University Teaching Hospital NHS Foundation Trust, North-west England Foundation School, UK

## Abstract

• This guide will signpost you to necessary resources, describe the pattern of work and day to day activities of an F2 doctor.
• It will briefly describe essential boxes to check for a successful programme completion and will look ahead for the post-F2 period.
• As an F2 standalone trainee, you will rotate through 3 different departments, and your job description might vary slightly in these units.

## 1 Introduction

The F2 standalone is a one-year programme run by the United Kingdom Foundation Programme Office (UKFPO), where eligible doctors are employed in the position of an F2 doctor and undertake clinical roles in various clinical settings, including acute and non-acute settings. The F2 standalone is equivalent to the second year of the 2-year UKFPO foundation programme as you have similar rotations, access to learning, and supervisors. On completion, you get a foundation programme certificate of completion.

I am an international medical graduate (IMG) F2 standalone trainee in the North West of England Foundation School and discovered very little first-hand information on the internet for IMGs starting the F2 standalone as their first NHS job. After consulting with my foundation school director, I developed this guide that will benefit people in this category. It may also help UK-trained F2 standalone trainees who have been out of foundation school and are returning to complete their foundation training. This guide will signpost them to changes in the curriculum that may have come in place since they took a break from training.

In summary, this guide will talk briefly about the F2 standalone application process, signpost you to necessary resources, describe the pattern of work and day to day activities of an F2 standalone doctor using my situation as a case study. Finally, it will briefly describe essential boxes to check for a successful programme completion as drawn from the curriculum and will look ahead for the post-F2 period.

## 2 Application process

The recruitment adverts, which the UKFPO nationally coordinates, come out in early January for posts starting in August of the same year, and applications are open for about two weeks. Applications are made using the Oriel (www.oriel.nhs.uk) platform.

**Fig. 1:**
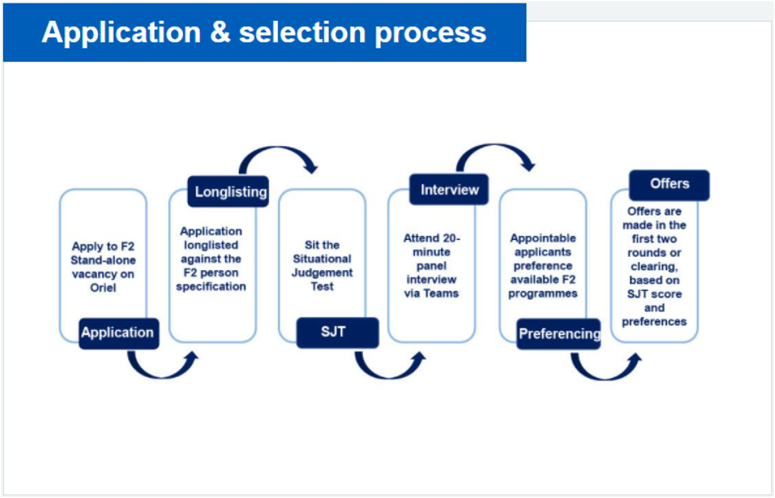
Image from Health Education England UKFPO official website: https://foundationprogramme.nhs.uk/programmes/f2-stand-alone/.

Fig. 1 from the UKFPO webpage summarises the application process. More detail on the application process is available in the Applicant Guidance for the same year. The application guidance for 2022 is referenced here [1]. It is outdated at the moment, and should only be studied for information. Kindly consult the application guidance in the year of application for updated information.

An important thing to note is that the English language requirement for the F2 standalone application is slightly higher than the one acceptable to the General Medical Council (GMC) for the Professional Linguistic Assessment Board (PLAB) examinations. The UKFPO requires a minimum of 7.5 in each of the listening, reading, writing and speaking parts of the academic version of the International English Language Test (IELTS) and a minimum of 400 in each of the listening, reading, writing and speaking parts of the Occupational English Test.

## 3 Pattern of work and tips

As an F2 doctor, you are fully registered with the GMC, and are expected to make patient management decisions appropriate for your level. Having at least one year of paid clinical experience in the form of a medical internship in your home country before commencing the post gives you the confidence to settle into the post.

As an F2 standalone trainee, you will rotate through 3 different departments, and your job description might vary slightly in these units. I am in the LIFT (Longitudinal Integrated Foundation Training) programme, meaning that I will work throughout the year in a General Practice (GP) surgery in addition to my three hospital-based rotations. The arrangement is to work for one week per month in the GP and the remaining three weeks in my hospital placement. The different hospital placements work out how this is spread across to ensure adequate exposure and full experience of the on-call rota.

I would either work standard days or on calls during my medicine posting. Standard days mean I will be on the ward from 9 a.m. to 5 p.m. On-calls might be weekend ward round covers, acute medicine unit shifts, medicine admissions from the emergency department (known as ED admissions) or night shifts.

My ward job involved reviewing the clinical status and investigation results of admitted patients, and creating a plan for them. I would usually discuss with the consultants/registrars before implementing my plans. In the medicine on-calls, I clerked referrals from the emergency departments or GPs and decided on a management plan. I would usually run it by a senior colleague if a case was confusing to me and required some senior input. In the initial four weeks, I did a lot of asking around, as I was new to the NHS system with so many things strange to me. I would encourage anyone starting out new in the system to ask as many questions and learn the most you can.

I noticed that taking locum shifts as allowed by the European Time Working Directive (EWTD) also helped me get faster exposure to a variety of clinical cases and the system as a whole. Reading through the clerking of my colleagues and seniors who trained in the UK also gave me an insight into how they document, and I applied a few things from them without losing my voice. The medical director once complimented my clerking during handover after an ED admissions night shift just three months into my medicine rotation-this was reassuring for me. After a shift, I would note down patients I had admitted and follow them up in a day to see how much the management plan I initiated differed from that of the consultant seeing the following day during the post-take round. This exercise also helped me improve significantly.

During my surgical posting, I would work standard days: ward work or theatre covers where I assist in surgeries; or calls (long days or night shifts), where I carry the orthopaedic SHO bleep. Carrying the on-call bleep was a little tricky initially, but I got the hang of it with time. I would take referrals or requests for orthopaedic advice from the wards, GPs, walk-in centres and the emergency department. In my first two weeks, most of the calls involved me gathering a history over the phone, seeing the patients, and immediately discussing with the registrar as I had very little orthopaedic exposure since finishing medical school. As time went on, I made some decisions by myself and reviewed the tricky ones with a senior.

From my experience, I would advise someone starting newly to consider taking a day or two of study leave from their first posting to shadow someone in the role they will be going to next, to mentally prepare for the new role and have a fair understanding of what is expected of you. This will help alleviate the anxiety of commencing a new post.

The GP sessions consist of a morning and afternoon clinic. During the morning clinic, I see the patients in 20 min and develop a plan. In the break between the clinics, I have some time to make referrals and write up scripts for the patients I saw in the morning clinic and then have my lunch and prepare for the afternoon clinic. In the afternoon clinic, I repeat the drill for the morning session, and I close for the day at 5 p.m. I usually discuss tricky cases with a senior GP at lunch break or the end of the afternoon clinic.

If you see a patient and are not sure what to do, the GPs are usually happy to be called upon to see the patient with you at any point during the clinic. As an F2 standalone doctor, you are in a training position and should not feel under any pressure to know everything.

## 4 Essential requirements

At the end of a completed F2 standalone programme, you should get a foundation programme certificate of completion (FPCC). This certificate demonstrates that you have achieved foundation competencies and is used to get into GP, speciality training or non-training jobs.

There is a list of checkboxes to tick during each of your rotations, starting as early as your first week in August. The guidelines for everything you need to do are embodied in the 2021 foundation programme curricullum [2]. I have linked an excellent easy to read summary [3] by a previous foundation doctor.

In this section of my brief guide, I will highlight some points in the new foundation programme curriculum, but reading this summary [3] will greatly benefit you.

### 4.1 Gathering evidence

You must record evidence gathered from all your placements in the eportfolio (Horus for England and Turas for Scotland, Wales and Northern Ireland). Whilst collecting the evidence, you should link this evidence to one or more of the 13 foundation programme capabilities [3]. These are 13 different competencies you have to exhibit in various clinical and non-clinical situations that tell the GMC and your appraisal body/foundation school that you are developing as a good clinician, a healthcare team worker and a professional.

When approaching an end of placement meeting with your supervisor, a vital thing is to start to write the summary narrative. The summary narrative is a short essay on each of the three higher learning outcomes [2,3]. These higher learning outcomes are the three main themes under which the 13 foundation programme capabilities exist. The higher level outcomes aim to characterise the foundation doctor as a:

1. Good clinician
2. Good healthcare team worker and
3. Professional

In the summary narrative essay, you should say why you have recorded the various pieces of evidence you have collected. This makes it easy for you and your supervisor to review your evidence during the meeting. In fact, this summary narrative is one of the foundation school's formative assessments at the Annual Review of Competency Progression meeting to decide if you meet the requirements to get an FPCC.

A list of the types of evidence the F2 doctor should collect

• Supervised learning events [3].
  o Direct observation of procedural skills (DOPS)
  o Mini-clinical encounter (Mini-CEX)
  o Case-based discussion (CBD)
  o Learning encounter and reflection note (LEARN)
• The personal learning log
• The summary narrative and reflections
• Placement supervision group-the clinical supervisor initiates this
• Team assessment of behaviour
• Clinical supervisor's reports
• Educational supervisor's reports

### 4.2 Requirements for a successful ARCP

• Completion of 12 months training (maximum permitted absence is 20 days other than annual leave)
• A satisfactory educational supervisor's end of year report
• A satisfactory clinical supervisor's end of placement report
• Satisfactory team assessment of behavior.
• Minimum of one satisfactory placement supervision group report
• Satisfactory completion of all curriculum outcomes-submit eportfolio evidence that you have met the 13 FPCs including life support skills required in FPC 2

### 4.3 After the F2 standalone

While in F2, you should be thinking of your next steps. Currently, most native graduates do an F3 or F4 year (third and fourth year post-graduation from medical school respectively, outside a structured training programme) where they take time out of training to work in Australia, New Zealand or locum in the UK. For IMGs, especially those whose right to remain in the United Kingdomare visa-bound, it is wise to decide on time what you want to do and work towards it at the start of the F2 year.

If you do not want to get into training, you could apply for service provision jobs. The positions usually open as early as February and March for jobs to begin in August after completing the F2 standalone post.

If you are interested in starting speciality or GP training after your F2 training, you must make your applications on Oriel in November, which is only three months after starting your F2 job. If you are gunning for a competitive specialty, requiring many tick boxes, you should start gathering the portfolio requirements even before starting the F2 standalone job.

## 5 Conclusion

It is an intense period to be honest and time goes very quickly. A lot of things are going on at once: you are trying to adjust to a new country, you are learning an entirely new healthcare system, you are ticking off F2 standalone portfolio requirements and also trying to gather the necessary portfolio for GP or specialty applications, amongst other family or personal commitments. It can feel very overwhelming, and I daresay a trust-grade job is easier for new entrants into the NHS, compared to the F2 standalone. Despite the difficulty of juggling all these in the F2 standalone year, I believe that putting one foot in front of the other and taking one day at a time will tide you through the process.

Try to connect with F2 standalone trainees in other trusts, as this will help keep you grounded and provide a sense of community. Making an effort to connect with the F2s in your trust will also pay off as they will help signpost you to courses or resources [4] available in your trust or deanery that you may not ordinarily learn about if you did not interact.

I wish you the very best in your endeavour! Have a wonderful F2 year!

## Ethical approval

Ethical approval for this article was not needed as the article was sourced from publicly available information and the authors personal experience.

## Sources of funding

No external funding was required for the production of this article.

## Author contribution

Ikechukwu Chukwudi is the sole author of the paper.

## Trial registry number

1. Name of the registry:
2. Unique Identifying number or registration ID:
3. Hyperlink to your specific registration (must be publicly accessible and will be checked):

## Guarantor

N/A.

## Consent

There were no patients or volunteers recruited in the execution of this article.

## Declaration of competing interest

There is no conflict of interest to declare.
